# Reactive oxygen species generated from skeletal muscles are required for gecko tail regeneration

**DOI:** 10.1038/srep20752

**Published:** 2016-02-08

**Authors:** Qing Zhang, Yingjie Wang, Lili Man, Ziwen Zhu, Xue Bai, Sumei Wei, Yan Liu, Mei Liu, Xiaochuan Wang, Xiaosong Gu, Yongjun Wang

**Affiliations:** 1 Key Laboratory of Neuroregeneration, Co-innovation Center of Neuroregeneration, Nantong University, Nantong 226001, PR China; 2 Department of Pathophysiology, School of Basic Medicine, Key Laboratory of Ministry of Education of China for Neurological Disorders, Tongji Medical College, Huazhong University of Science and Technology, Wuhan 430030, PR China

## Abstract

Reactive oxygen species (ROS) participate in various physiological and pathological functions following generation from different types of cells. Here we explore ROS functions on spontaneous tail regeneration using gecko model. ROS were mainly produced in the skeletal muscle after tail amputation, showing a temporal increase as the regeneration proceeded. Inhibition of the ROS production influenced the formation of autophagy in the skeletal muscles, and as a consequence, the length of the regenerating tail. Transcriptome analysis has shown that NADPH oxidase (NOX2) and the subunits (p40^phox^ and p47^phox^) are involved in the ROS production. ROS promoted the formation of autophagy through regulation of both ULK and MAPK activities. Our results suggest that ROS produced by skeletal muscles are required for the successful gecko tail regeneration.

Several adult vertebrates possess the remarkable ability to regenerate the lost appendages, such as limbs and tails^1^,^2^,^3^. A large number of investigations demonstrate that appendage regeneration in fishes, amphibians and reptiles shares conserved fundamental regeneration stages, and more or less, the distinct cellular and molecular mechanisms^4^,^5^,^6^,^7^. Amputation of appendage results in the wound healing, followed by the formation of a blastema, in which the progenitor cells will eventually differentiate to the various tissues including muscle and cartilage, etc. Cell fate transformation involved in dedifferentiation or differentiation occurs incessantly through alterations of both cytoplasmic constituents and nuclear gene expression^8^. The injury-induced signals are spatially and temporarily activated to trigger and drive such regenerative programs^9^,^10^,^11^.

Tissue damage produces reactive oxygen species (ROS) largely by non-inflammatory wound-resident cells, as shown in tadpole tail amputation and zebrafish epidermal wounding^11^,^12^. Despite their toxic potential, growing evidence indicates that increased ROS production acts important roles in the regulation of signal pathways that are required to promote appendage regeneration^11^,^13^. Skeletal muscle is one of tissue sources to produce high rates of ROS under pathological conditions known as oxidative stress^14^,^15^,^16^,^17^. It has been found that a moderate increase of ROS in skeletal muscle can lead to cellular adaptation and protection against stresses. But, sustained high levels of ROS may result in the induction of autophagy, an evolutionarily conserved degradative pathway by which intracellular proteins and organelles are delivered to the lysosome for destruction and recycling^16^,^18^,^19^. Autophagic degradation has been associated with important physiological functions including developmental and differentiation processes of animals, in addition to its roles in myopathies^20^,^21^. Defective autophagy has been found in some congenital muscular dystrophies, and activation of autophagy is able to restore myofiber survival and ameliorate the dystrophic phenotype^21^. As such, autophagy induced by ROS of skeletal muscle is essential for cellular survival and cell fate transformation, which might affect appendage regeneration.

As a self-degradative process to balance sources of energy in development and in response to nutrient stress, autophagy is regulated by the energy sensor AMP activated protein kinase (AMPK) and the amino-acid sensor mammalian target of rapamycin complex I (mTORC1), followed by the activation of the ULK1-ATG13-FIP200 complex^19^. Also, it can be induced by activation of MAPKs signaling pathway^22^,^23^. However, the mechanism of ROS contributing to the tail regeneration through induction of autophagy remains unknown. In the present study, we observed the ROS production *in vivo* using a free permeable radical sensor around the injured sites following gecko tail amputation. We subsequently evaluated their effects on the tail regeneration by chemical inhibition, and further examined the associated signaling pathway by transcriptional sequencing analysis. Our results revealed that ROS in the injured skeletal muscles were required for the successful regeneration of tail by inducing formation of autophagy.

## Results

### ROS generated by skeletal muscle were involved in the tail regeneration

**Figure 1:**
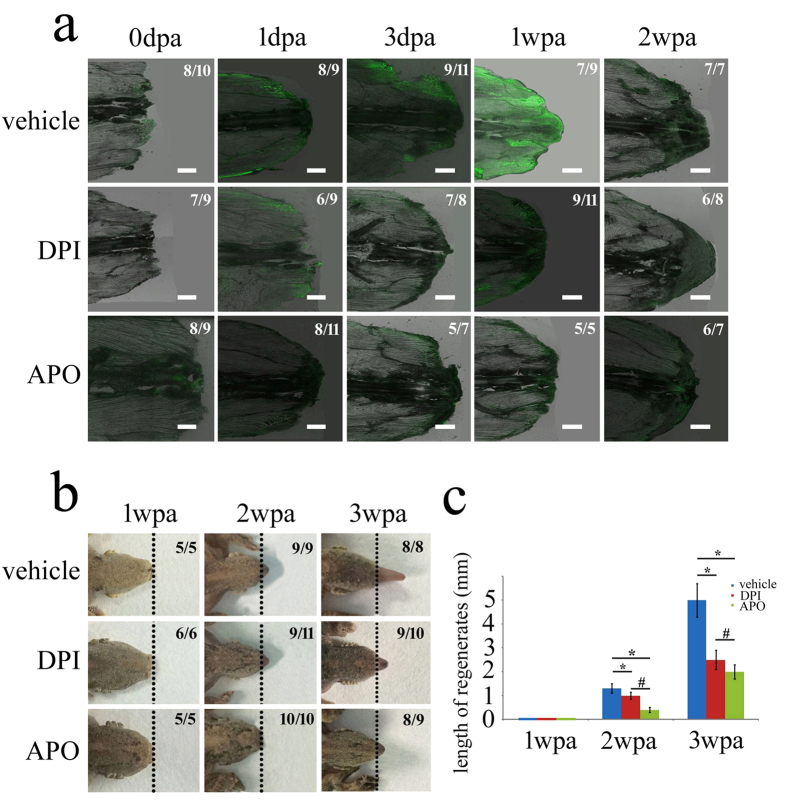
Production of ROS during gecko tail regeneration and the effects of inhibitors on the regenerates. (**a**) Amputation-induced ROS were detected with a fluorescent probe (H_2_DCFDA) before or after treatment with ROS inhibitors DPI and APO, respectively; (**b**) The effects of ROS inhibitors DPI and APO on the tail regeneration; (**c**) Statistic analysis of regenerating tails. The ratio in the right upper indicates the statistical samples out of the whole numbers. Data are expressed as mean ± SEM; *p < 0.05 *versus* vehicle; ^#^p < 0.05 between different ROS inhibitors. Scale bars, 400 μm in (**a**).

To understand the role of ROS during the gecko tail regeneration, we firstly detected the production of ROS in the amputated tail *in vivo*, using a free permeable radical sensor H_2_DCFDA, which could be converted to the fluorescent 2′, 7′-dichlorofluorescein (DCF) upon cleavage of the acetate group through oxidation^11^,^13^. Fluorescence was detected from 520 to 583 nm, a wavelength range beyond autofluorescence of gecko tissues. Low levels of ROS have been observed under the epidermis at 1 dpa, while ROS production has been shown to be significantly increased in the outer area of skeletal muscle at 3 dpa (Fig. 1a). The high rates of ROS production were found in all the skeletal muscle around the injured site at 7d regenerates (Fig. 1a). The data indicate that gecko tail amputation induces a substantial production of ROS in the skeletal muscle.

We next used 50 μM diphenyleneiodonium (DPI), a flavoprotein inhibitor, which targets the NOX subunit, and 600 μM apocynin (APO), which disrupts the assembly of the NOX complex^11^, to reduce the ROS production during the tail regeneration (Fig. 1a). Lowering the ROS levels by continual treatment of the inhibitors significantly attenuates gecko tail regeneration, as evidenced by shorter tail length at 2 weeks and 3 weeks post-amputation (Fig. 1b,c). Comparatively, the APO inhibitor is more efficient than DPI in reducing the production of ROS. The results indicated that ROS generated by skeletal muscle were involved in the tail regeneration.

### Inhibition of ROS damaged muscles by preventing formation of autophagy

**Figure 2:**
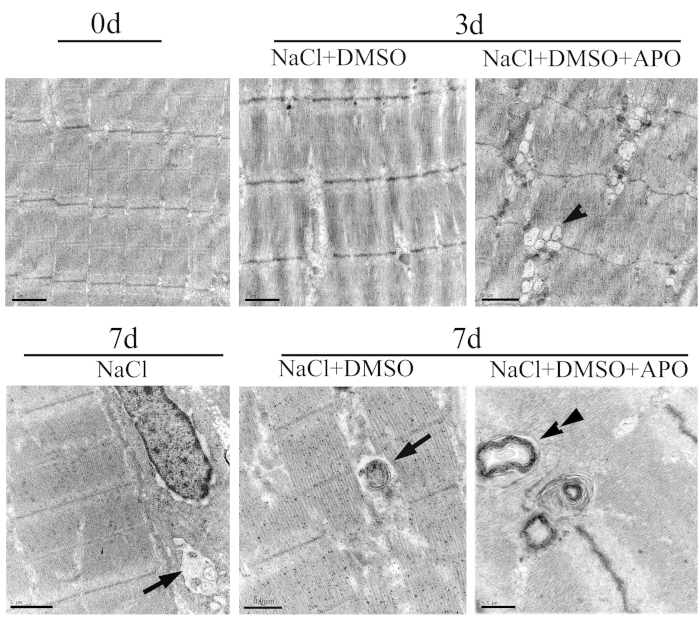
The ultrastructure of organelles in the skeletal muscle using transmission electron microscopy following treatment of the regenerate with or without 600 μM APO (n = 6). About 1 ~ 2 autophagic vesicles or myeloid bodies were observed in each visual field from 6 replicates following drug treatment at 7 dpa. Arrowhead indicates vacuoles. Arrow indicates autophagic vesicles; Tandem arrowhead indicates myeloid bodies. Scale bars, 0.5, 0.9 or 1 μm was marked in the photographs.

To evaluate the effects of ROS reduction on the skeletal muscles, we firstly examined the ROS-inhibited myofibers by light microscopy at 3 dpa and 7 dpa. The results showed that inhibition of ROS led to a decrease of the cross-sectional area of the myofibers at 7 dpa (see Supplementary Fig. S1). We further detected the ultrastructural organelles of muscles using transmission electron microscopy following treatment of the regenerate with 600 μM APO. Obviously, inhibition of ROS resulted in the structural aberrations of myofibers. At 3d following APO treatment, the Z-discs of skeletal muscle are misaligned, fragmented, and irregularly spaced. The mitochondria were vacuolated (Fig. 2). Of note, the formation of autophagic vacuoles in the skeletal muscle of 7d control group was prevented by APO treatment. Instead, the myeloid bodies formed (Fig. 2). The saline group was also detected to exclude the effects of DMSO on the formation of autophagic vacuoles. The data indicated that amputation-induced ROS were involved in the formation of autophagy in skeletal muscles.

### Functional annotations of ROS-related genes in the regenerating skeletal muscles

**Figure 3:**
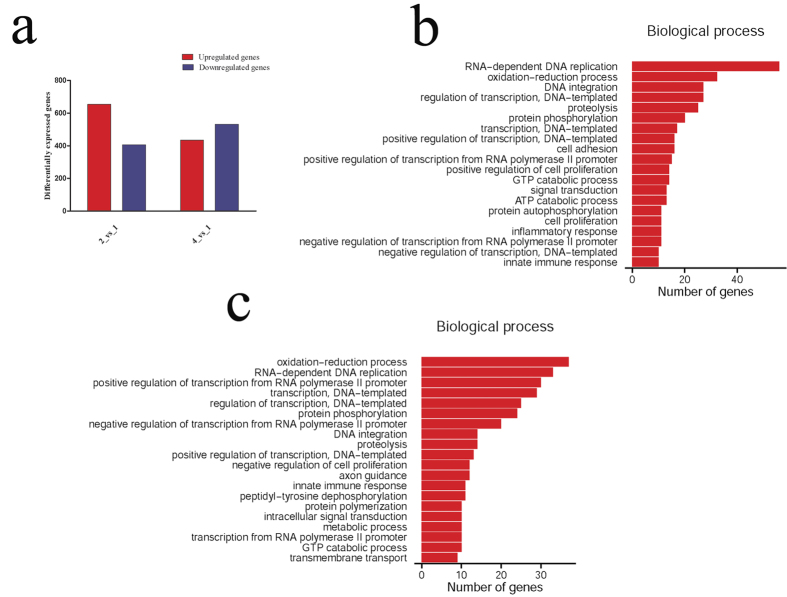
Functional annotations of differentially expressed genes in the regenerating skeletal muscles. (**a**) Bar graphs of differentially expressed genes at 3 dpa and 7 dpa, respectively; (**b**,**c**) Most significantly enriched groups for the differentially regulated mRNAs relating to biological processes at 3 dpa (**b**) and 7 dpa (**c**).

**Figure 4:**
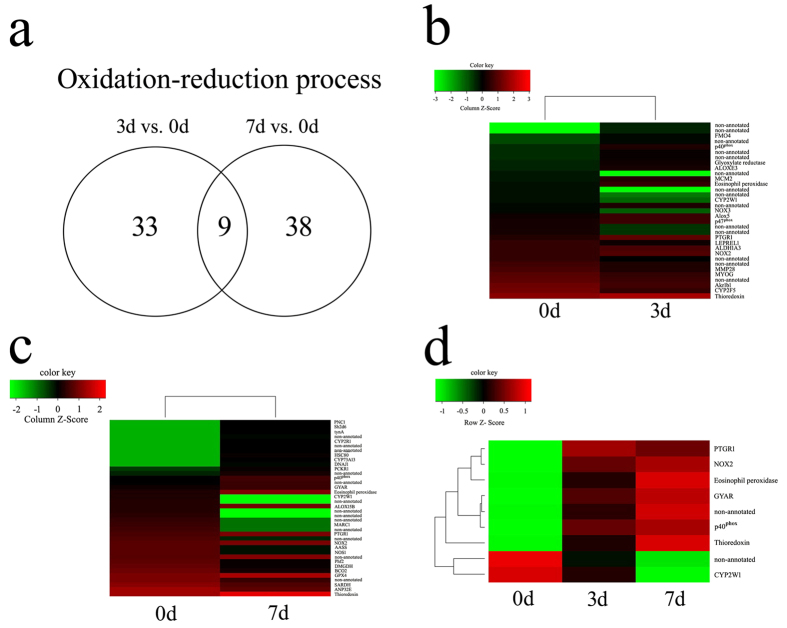
Identification of ROS-related NADPH oxidase(s) following gecko tail amputation. (**a**) Schematic diagram of the number of differentially expressed genes involved in the oxidation-reduction process at 3 dpa and 7 dpa, respectively; (**b,c**) Heatmap and cluster dendrogram of oxidation-reduction related genes at 3 dpa (**b**) and 7 dpa (**c**), respectively; (**d**) Heatmap and cluster dendrogram of integrated oxidation-reduction related genes at 3 dpa and 7 dpa.

To ascertain ROS production-related genes in the regenerating skeletal muscles, we performed transcriptome analysis on skeletal muscles at 0d, 3d and 7d following tail amputation. A total of 1058 (3d *versus* 0d) and 966 (7d *versus* 0d) differentially expressed mRNAs were identified with defined criteria of P < 0.05 and a greater than twofold or less than twofold changes (Fig. 3a). GO analysis reveals that oxidation-reduction process, which is related to the ROS production^24^, is significantly functional annotated, together with other biological processes including RNA-dependent DNA replication, DNA integration, regulation of transcription, proteolysis, etc. (Fig. 3b,c). At 3 dpa, 33 differentially expressed genes are involved in the oxidation-reduction process, among which ROS-related NADPH oxidase 2 (NOX2) and its subunits (p40^phox^, p47^phox^) have been identified. Another NOX homolog, NOX3, was excluded from the potential candidate of ROS-related player for its lower abundance. The hierarchical cluster analysis demonstrated that the expression of these genes were significantly upregulated in the skeletal muscles following tail amputation, suggesting their roles on ROS production (Fig. 4a,b). While at 7 dpa, a total of 38 differentially expressed genes participate in such process, and NOX2 and p40^phox^ have been shown to be upregulated (Fig. 4a,c). The data indicate that NOX2 and p40^phox^ are involved in the regulation of sustained generation of ROS, and p47^phox^ plays a role only in the shorter time (3 dpa) (Fig. 4a,d). In addition, the activities of anti-oxidative enzymes including Thioredoxin (THIO), Glyoxylate reductase (GYAR) and Prostaglandin reductase 1 (PTGR1) have been found to elevate concomitantly, suggesting a protective role of muscles in response to excessive production of ROS (Fig. 4b–d).

**Figure 5:**
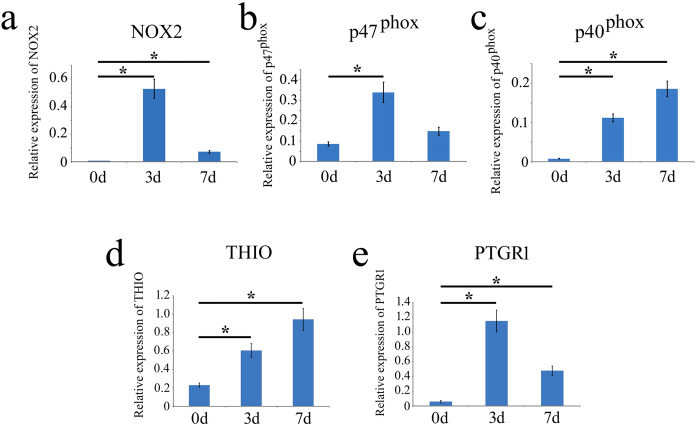
Real-time PCR analysis of oxidation-reduction related genes. (**a–c**) Transcriptional analysis of NADPH oxidase 2 (*NOX2*) and its subunits Neutrophil cytosol factor 4 (*p40*^*phox*^) and NADPH oxidase organizer 2 (*p47*^*phox*^) in the skeletal muscles at 0 dpa, 3 dpa and 7 dpa, respectively. (**d,e**) Transcriptional analysis of anti-oxidant genes Thioredoxin (*THIO*) and Prostaglandin reductase 1 (*PTGR1*). Error bars represent the standard deviation (p < 0.05).

Quantitative real-time PCR was further performed to substantiate the results from transcriptome analysis. The results demonstrated that the expression of ROS-related genes, *NOX2, p40*^*phox*^ and *p47*^*phox*^, as well as anti-oxidant genes, *THIO* and *PTGR1*, displayed a consistency with those of transcriptome analysis of skeletal muscles at 0 dpa, 3 dpa and 7 dpa, respectively (Fig. 5a–e). The data indicate that NADPH oxidases play important roles in the ROS production during the gecko skeletal muscle regeneration.

### Identification of ROS-mediated signaling in the regulation of skeletal muscle regeneration

**Figure 6:**
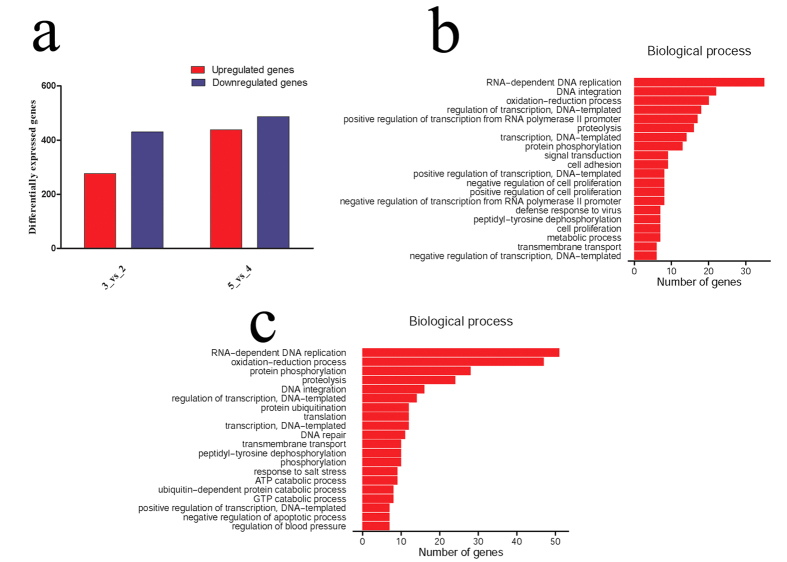
Functional annotations of differentially expressed genes in the regenerating skeletal muscles following ROS inhibition. (**a**) Bar graphs of differentially expressed genes at 3d and 7d following APO inhibition; (**b,c**) Most significantly enriched groups for the differentially regulated mRNAs relating to biological processes at 3d (**b**) and 7d (**c**) following APO inhibition.

**Figure 7:**
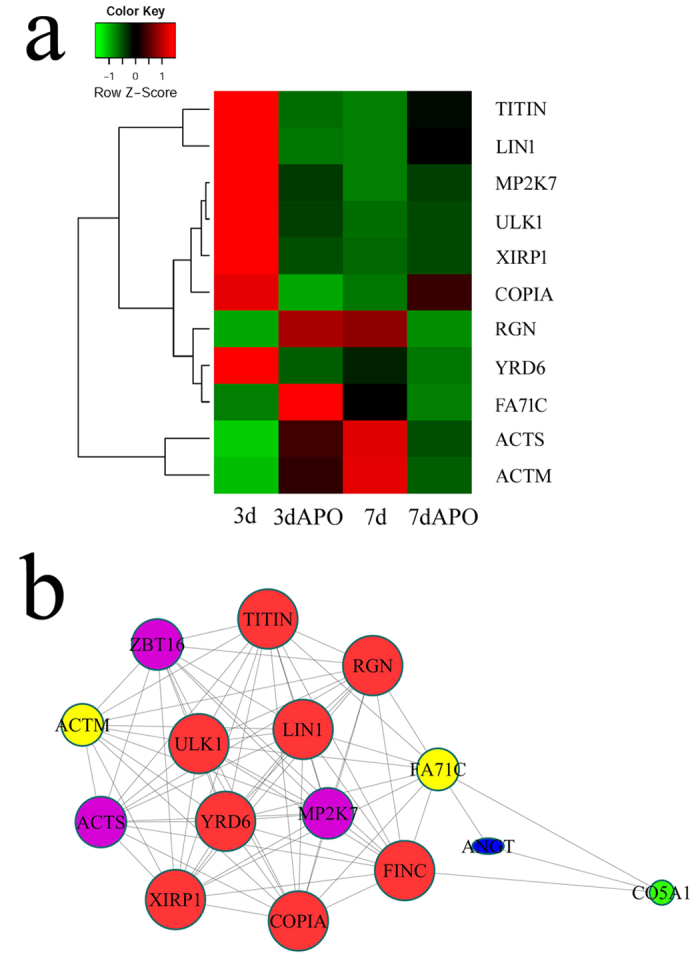
Identification of misregulated autophagy-related genes following APO inhibition. (**a**) Heatmap and cluster dendrogram of integrated differentially expressed genes at 3d and 7d following APO inhibition; (**b**) Integrated regulatory network of the differentially expressed genes.

To unveil ROS-mediated signaling in the regulation of skeletal muscle regeneration, we analyzed the transcriptomes of the skeletal muscle of the regenerates, following treatment with 600 μM APO for 3d and 7d. A total of 707 (3d APO *versus* 3d control) and 916 (7d APO *versus* 7d control) differentially expressed genes were identified (Fig. 6a). GO analysis has shown that several important biological processes including RNA-dependent DNA replication, oxidation-reduction, proteolysis, protein phosphorylation, etc. were significantly functionally annotated, indicating ROS inhibition-related effects (Fig. 6b,c). Integration of differentially expressed genes of 3d APO *versus* 3d control with those of 7d APO *versus* 7d control, characterized 11 functional genes associated with 7 important biological processes. These genes displayed dynamic alteration following APO treatment, and also showed a correlation between each other, as shown in the gene regulatory network (Fig. 7a,b). Interestingly, the expression levels of uncoordinated-51 (unc-51)-like kinase 1 (ULK1) and mitogen-activated protein kinase kinase 7 (MP2K7), which are involved in the regulation of autophagy, were decreased after APO treatment. But the expression of XIRP1, a marker of skeletal muscle damage severity, was significantly increased^25^. These data suggest that some protein kinases involved in autophagy, are downregulated following inhibition of ROS in skeletal muscles.

### ROS regulation of autophagy in skeletal muscles of gecko regenerates

**Figure 8:**
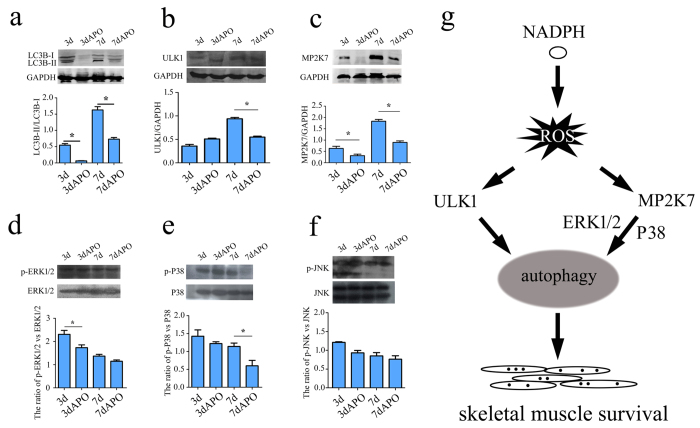
ROS mediate autophagy in the skeletal muscle through regulation of both ULK and MAPK activities. (**a**) Western blot showing autophagy markers LC3 I-II in the skeletal muscle following tail amputation; (**b–f**) Western blot of ULK1, MP2K7, and phosphorylated ERK1/2, p38 and JNK kinases; (**g**) Illustration of ROS regulation on the autophagy-related pathway. Error bars represent the standard deviation (p < 0.05).

To verify whether ROS were implicated in the regulation of autophagy in skeletal muscles of the regenerates, we firstly measured the levels of the free (LC3B-I) and the lipidated (LC3B-II) forms of LC3B protein. LC3B-II represents the portion of the cellular LC3B protein pool that is incorporated into autophagosome membranes^19^. During the tail regeneration, LC3B-II/LC3B-I ratio increased temporally (Fig. 8a), indicating that autophagy is required for the skeletal muscles regeneration. The formation of the autophagosome requires the upregulation of several autophagy-related proteins. We then measured the protein contents of ULK1 and MP2K7, and an increase of ULK1 and MP2K7 protein levels was observed at 7 dpa (Fig. 8b,c). The slight differences between the transcriptional and translational levels imply regulatory diversity of the kinases (Fig. 7a). APO treatment of the amputated stump significantly decreased the protein levels of both ULK1 and MP2K7, suggesting that the autophagy was under regulation of these kinases (Fig. 8b,c). Accordingly, the levels of phosphorylated ERK1/2 and p38, the kinases downstream of MP2K7, were decreased following ROS inhibition at 3 dpa (p-ERK1/2) and 7 dpa (p-p38), while the level of phosphorylated JNK was not affected (Fig. 8d–f). The reduction of ROS eventually led to the growing damage of the muscle, as shown by the increase of XIRP1 (Fig. 7). The data indicate that ROS-regulated autophagy in skeletal muscles is beneficial for the tail regeneration.

## Discussion

NOX-derived ROS are produced in almost every tissue to exert various physiological and pathological functions, including host defense and anti-inflammation, cellular signaling, regulation of cellular death and senescence, and pathogenesis of specific tissues and organs^24^. Nerves have been shown to control tissue redox levels in mature tissue, and also modify ROS levels induced by wounding and amputation^26^. Recently, injury-induced ROS production has been found to be an important regulator in *Xenopus* tadpole tail, zebrafish fin and planarian regeneration through activation of cell proliferation^11^, induction of cell apoptosis^13^ or interference with early neoblast differentiation^27^. Interestingly, ROS also regulate tail regeneration of gecko, the non-mammalian amniote, suggesting a phylogenetically conserved function of ROS signaling in the regulation of appendage regeneration. ROS production has been shown to promote cell proliferation through activation of Wnt/β-catenin signaling^11^, or induction of apoptosis and JNK signaling for compensatory cell proliferation^13^. The transcriptome analysis of injured gecko skeletal muscle also identified the proliferative pathway in relation to the ROS production. Whether the production of ROS is involved in the cellular events of satellite cells, which lie beneath the basal lamina of the original muscle fiber, deserves further study.

Growing evidence has shown that the NADPH oxidases are likely to be the major superoxide generating sources in contracting skeletal muscle^28^,^29^. In the present study, both DPI and APO, the inhibitors of NADPH oxidases, efficiently decreased the production of ROS following gecko tail amputation, substantiating such conclusion. To date, a total of seven homologs of NADPH oxidases have been found, including NOX1, NOX2, NOX3, NOX4, NOX5, DUOX1 and DUOX2. These enzymes display a distinct tissue distribution. NOX2, originally recognized as phagocyte NADPH oxidase, has also been found to exist in many other cell types, including fibroblasts, various tumor cells and skeletal muscle^24^. The enzyme binds with membrane subunit p22^phox^ and cytosolic subunits p40^phox^, p47^phox^ and p67^phox^ to generate superoxide and downstream ROS. Using trancriptome sequencing, we identified NOX2 and its two cytosolic regulatory subunits: p40^phox^ and p47^phox^, being upregulated in the skeletal muscle during the gecko tail regeneration, suggesting the critical role of tissue-specific NOX members in the ROS production.

ROS were widely assumed to be cytotoxic to skeletal muscle fibres. However, recent findings indicate that increased ROS production plays important roles in the regulation of signaling pathways that are required to promote skeletal muscle adaptation^16^. For example, a moderate increase of ROS production in skeletal muscle is beneficial for the protection against future stresses. In contrast, high levels of ROS production may promote proteolysis that potentially associates with autophagy, a major catabolic pathway by which eukaryotic cells degrade and recycle macromolecules and organelles^16^,^30^. ROS oxidize and inhibit Atg4, a protease responsible for microtubule associated protein (MAP) light chain 3 (LC3) delipidation, promoting the autophagosome maturation^30^. ROS-induced autophagy is often linked to muscle atrophy, disuse and aging. However, recent evidence has shown that activation of autophagy is able to rescue myofiber degeneration^21^. Also, autophagy protects against cellular apoptosis and is required for myoblast differentiation^31^. Therefore, autophagy is now thought of as a survival mechanism in the skeletal muscle. In the present study, we have shown that autophagy induced by ROS in the skeletal muscle is involved in the tail regeneration, consistent with its essential role in zebrafish caudal fin regeneration, suggesting a novel cellular function of the autophagic process in the appendage regeneration.

Autophagy induction is controlled by complex regulatory mechanisms involving in multiple signals, including nutrients, ROS, hypoxia, accumulation of misfolded proteins, etc.^20^. Most signals converge at the level of the mTORC1. In condition of signals present, class I phosphatidylinositol-3-kinase (PIK3C1) activates mTORC1, which inhibits autophagy by binding and phosphorylating ULK1 or ULK2 and Atg13 within the ULK complex^32^. In contrast, repression of mTORC1 makes it dissociate from the ULK complex, promoting ULK activity and FIP200 hyperphosphorylation, thus activating autophagy^33^. MAPK signaling has also been shown to play an important role in the regulation of autophagy, such as influencing autophagic activity and controlling autophagy process at the maturation step^8^,^34^. Following gecko tail amputation, inhibitors of ROS interfered with the formation of autophagy in the skeletal muscle through regulation of both ULK and MAPK activities, suggesting diverse mechanisms of ROS signaling in regulation of autophagy in the skeletal muscle during the tail regeneration.

In conclusion, as illustrated in Fig. 8g, NOX2 and its two cytosolic regulatory subunits: p40^phox^ and p47^phox^, are involved in the production of ROS in the skeletal muscle following gecko tail regeneration. The increased ROS promote the formation of autophagy through regulating both ULK and MAPK activities. ROS produced by skeletal muscles are required for the successful gecko tail regeneration.

## Methods

### Animals

Adult *Gekko japonicus* were used as described by Wang *et al.*^35^. Briefly, adult animals were fed *ad libitum* with mealworms and housed in an air-conditioned room with a controlled temperature (22–25 °C) and saturated humidity. Anesthesia was induced by cooling the animals on ice prior to tail amputation. Amputation was performed at the sixth caudal vertebra, identified based on the special tissue structure present at that position^36^, by placing a slipknot of nylon thread and pulling gently until the tail was detached, thus mimicking the autotomy undergoing for natural defense.

All experiments were conducted in accordance with guidelines established by the NIH, found in *Guide for the Care and Use of Laboratory Animal* (1985), and by the Society for Neuroscience, found in *Guidelines for the Use of Animals in Neuroscience Research*. The experiments were approved according to the Animal Care and Use Committee of Nantong University and the Jiangsu Province Animal Care Ethics Committee. All geckos (n = 20) were anaesthetized on ice prior to euthanatizing.

### Reactive oxygen species (ROS) detection

The compound 2′, 7′-dichlorofluorescin diacetate (H_2_DCFDA, Calbiochem) was used to monitor the accumulation of ROS in the amputated gecko tail. Fluorescent DCF was formed through ROS oxidation. Gecko tail stump was immersed in an eppendorf tube containing the 50 mM H_2_DCFDA solution for 30 min, following tail amputation. The regenerates were collected at desired stages, cut into section of 100 μm, and imaged under two-photon laser scanning fluorescence microscopy (Leica sp5). Fluorescence was excited with a 488 nm laser and detected from 520 to 583 nm.

### Drug treatments

The tail stump was immersed in an eppendorf tube containing 50 μM diphenyleneiodonium (DPI, Sigma-Aldrich) or 600 μM apocynin (APO, Sigma-Aldrich) in 0.1% dimethylsulphoxide (DMSO, Sigma-Aldrich) saline solution for 6 h, then 5 μl of 50 μM DPI or 600 μM APO were injected intraperitoneally every two days. Control experiments were performed according to the same protocol, with an equivalent amount of vehicle.

### Transmission electron microscopy

The dissected muscles were immersion-fixed in fixative (1% glutaraldehyde, 4% formaldehyde in 0.1M sodium phosphate at pH 7.3) for 1 h, and treated with 1% osmium for 1 h, stained en bloc with saturated aqueous uranyl acetate for 1 h, dehydrated, and embedded in Epon. Ultra-thin sections from the selected areas were contrasted with uranyl acetate and lead citrate and viewed using an FEI Tecnai^TM^ 12 transmission electron microscope at 120 kV. Images were digitally captured.

### Quantitative real-time polymerase chain reaction (Q-PCR)

Total RNA was prepared with mirVana miRNA Isolation Kit (Ambion, Austin, TX) from skeletal muscles of 20 geckos amputated from the sixth caudal vertebra at 0 day, 3 days and 1 week, respectively. For Q-PCR examination of transcriptional expression of related genes, the first-strand cDNA was synthesized using Omniscript Reverse Transcription Kit (QIAGEN) in a 20 μL reaction system containing 2 μg total RNA, 0.2 U/μL M-MLV reverse transcriptase, 0.5 mM dNTP mix, 1 μM Oligo-dT primer. The cDNA was diluted 1:5 before use in Q-PCR assays. Q-PCR reactions were performed in a final volume of 10 μl according to the manual (Roche). The Rotor-Gene 5 software (Corbett Research, Rotor-Gene, Australia) was used for real-time PCR analysis. Fluorescence was recorded during each annealing step. At the end of each PCR run, data were automatically analyzed by the system and amplification plots obtained. *NOX2* full-length plasmid was used to prepare standard curves and used as a specificity control for real-time PCR. The expression levels were normalized to endogenous reference gene, which was validated by geNorm and Normfinder analyses from potential reference genes *EF-1α, GAPDH* and 18S RNA. In addition, a negative control without the first-strand cDNA was also performed. Gene expression analyses were performed with MIQE guidelines taken into account^27^. Details of the procedure are described in Supplementary Table1.

### Sequencing of mRNA

Total RNA of samples following treatment with 600 μM APO or vehicle for 0 day, 3 day and 1 week respectively, was extracted from skeletal muscles of tail stump using the mirVana miRNA Isolation Kit (Ambion, Austin, TX) according to the manufacturer’s instructions. They were then selected by RNA Purification Beads (Illumina, San Diego, CA), and undergone library construction and RNA-seq analysis. The library was constructed by using the Illumina TruSeq RNA sample Prep Kit v2 and sequenced by the Illumina HiSeq-2000 for 50 cycles. High-quality reads that passed the Illumina quality filters were kept for the sequence analysis.

### Bioinformatics Analysis

Differentially expressed mRNA was designated in criteria of greater than 2-fold or less than 2-fold change in comparison with control. Function of genes was annotated by Blastx against the NCBI database or the AGRIS database (http://arabidopsis.med.ohio-state.edu/downloads html) with E-value threshold of 10^−5^. Gene ontology (GO) classification was obtained by WEGO (http://wego.genomics.org.cn/cgi-bin/wego/index.pl) via GO id annotated by Perl and R program. Kyoto Encyclopedia of Genes and Genomes (KEGG) pathways were assigned to the sequences using KEGG Automatic Annotation Server (KAAS) online. For all heatmaps, genes were clustered by Jensen-Shannon divergence.

### Western Blot Analysis

Protein extracts were prepared from skeletal muscles. Equal amounts of protein were subjected to SDS-PAGE and electrotransferred to PVDF membrane (Bio-Rad, Hercules, CA). The membrane was blocked with 5% non-fat dry milk in Tris-HCl buffered saline, pH 7.4 with Tween-20 and incubated with the primary antibody to detect LC3B-I/LC3B-II (Cell Signaling Technology), ULK1 (Santa Cruz Biotechnology), MP2K7 (Abbexa), ERK1/2, p-ERK1/2, p38, p-p38, JNK, p-JNK (Cell Signaling Technology), according to the manufacturer’s recommendations. Antibody binding was detected by HRP-conjugated species-specific secondary antibody followed by enhanced chemiluminescence (Pierce Chemical Company, Rockford, IL).

### Statistical Analysis

Statistical significance of differences between groups was analyzed by one-way analysis of variance (ANOVA) followed by Bonferroni’s post-hoc comparisons tests with SPSS 15.0 (SPSS, Chicago, IL, USA). Normality and homoscedasticity of the data were verified before any statistical analysis using levene’s test. Statistical significance was set at p < 0.05.

## Additional Information

**How to cite this article**: Zhang, Q. *et al.* Reactive oxygen species generated from skeletal muscles are required for gecko tail regeneration. *Sci. Rep.* **6**, 20752; doi: 10.1038/srep20752 (2016).

## Supplementary Material

Supplementary Information
