# Supplementary material for: Reactive oxygen species generated from skeletal muscles are required for gecko tail regeneration
Source: Sci Rep. 2016 Feb 8;6:20752. doi: 10.1038/srep20752 (PMC4745102; doi:10.1038/srep20752)
Supplement: Supplementary Information [file srep20752-s1.pdf]

Reactive oxygen species generated from skeletal muscles are required for gecko tail regeneration

Qing Zhang[1,2], Yingjie Wang[1], Lili Man[1], Ziwen Zhu[1], Xue Bai[1], Sumei Wei[1], Yan Liu[1], Mei Liu[1], Xiaochuan Wang[2], Xiaosong Gu[1], Yongjun Wang[1]*

[1]Key Laboratory of Neuroregeneration, Co-innovation Center of Neuroregeneration, Nantong University, Nantong 226001, PR China

[2]Department of Pathophysiology, School of Basic Medicine, Key Laboratory of Ministry of Education of China for Neurological Disorders, Tongji Medical College, Huazhong University of Science and Technology, Wuhan 430030, PR China

[*]To whom correspondence should be addressed. Email: wyjbs@ntu.edu.cn. Fax: 0086-513-85511585. Tel: 0086-513-85051818.

Key Laboratory of Neuroregeneration, Nantong University, 19 Qixiu Road, Nantong 226001, P.R. China

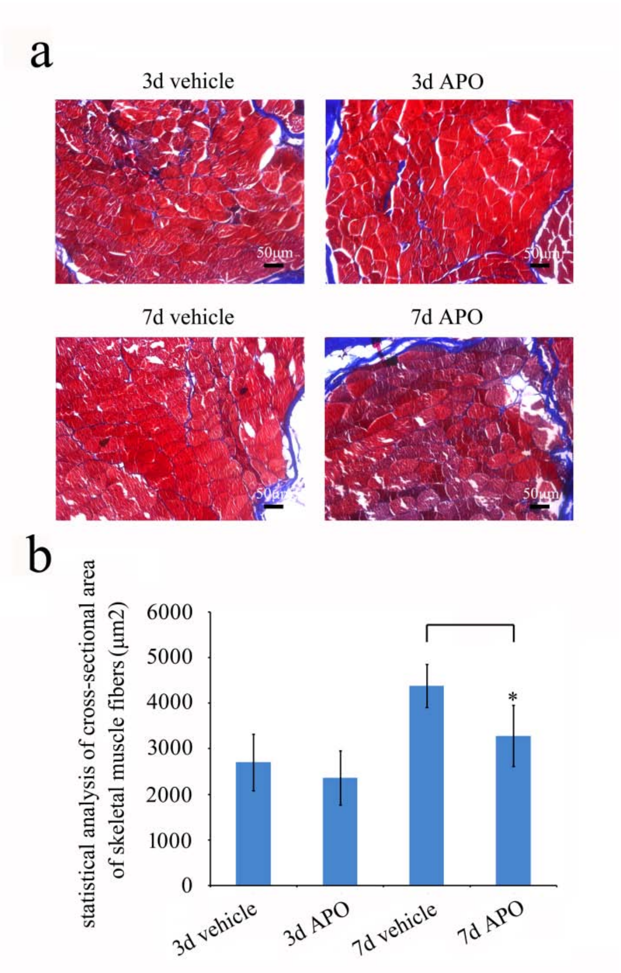

FigureS1. Masson's trichrome staining of gecko skeletal muscle following APO treatment of the amputated tail

Table S1: MIQE guidelines concerning qPCR experiment

**Experimental design**	
Definition of experimental and control groups	Experimental groups:<br>- Regenerating geckos (skeletal muscles) with the tail stump immersed in 600 µM APO in 0.1% DMSO for 6 h, and then injected with 5 µl of 600 µM APO intraperitoneally every two days.<br><br>Control groups:<br>- Regenerating geckos (skeletal muscles) were treated with 0.1% DMSO in parallel.
Number within each group	n ≥ 20
**Sample**	
If frozen, how and how quickly?	Snap frozen in liquid nitrogen
Sample storage conditions and duration	Stored at -80 °C for one month maximally
**Nucleic acid extraction**	
Procedure and/or instrumentation	mirVana miRNA Isolation Kit (Ambion, Austin, TX)
Nucleic acid quantification	RNA concentrations were assessed using spectrophotometry
Instrument and method	Nanodrop ND-1000 spectrophotometer (NanoDrop Technologies)
Purity	260/280 and 260/230 analysis
**Reverse transcription**	
Complete reaction conditions	Omniscript Reverse Transcription Kit (QIAGEN)
Amount of RNA and reaction volume	2 µg of total RNA in a reaction volume of 20 µl
Storage condition of cDNA	cDNA was 1/5 diluted in water before storage at -20°C
**qPCR target information**	
Gene symbol and sequence accession number	*NOX2*, *$P40^{phox}$*, *$P47^{phox}$* , *THIO*, *PTGR1* *EF-1α* and *GAPDH* are defined by transcriptome sequencing
In silico specificity screen (BLAST)	*NOX2* (NADPH oxidase 2); *$P40^{phox}$* (Neutrophil cytosol factor 4); *$P47^{phox}$* (NADPH oxidase organizer 2); *THIO* (thioredaxin); *PTGR1* (prostaglandin reductase 1); *EF-1α* (elongation factor-1α) ; *GAPDH* (Glyceraldehyde 3-phosphate dehydrogenase)
**qPCR oligonucleotides**	

<table>
<tr><td rowspan="7">Primer sequences</td><td colspan="2">NOX2</td><td>5′-CCA TTG AGC TCC AGA TGA AGA A-3′ (F)
5′-CAG TCC CCT ACA ATA CGT ATG TG-3′ (R)</td></tr>
<tr><td colspan="2">P40^{phox}</td><td>5′-CTA CTT AAC CTG CCT GCC TG-3′ (F)
5′-TTC TGT CAA AGC CAG GTT CC-3′ (R)</td></tr>
<tr><td colspan="2">P47^{phox}</td><td>5′-TCC CGA CAC CCT ACA TCA TC-3′ (F)
5′-TCA TGC CGT CCT CAC TAA GA-3′ (R)</td></tr>
<tr><td colspan="2">THIO</td><td>5′-CCC ACA GGA AAT AAA CAA TGG AAA A-3′ (F)
5′-AGG AGG AAG ACC ACA TGA AAC AA-3′ (R)</td></tr>
<tr><td colspan="2">PTGR1</td><td>5′-GAA AGC ATC TCC CAA TGG TTA CG-3′ (F)
5′-ATA GCA CCA CAT ACT GCA ATC CTC C-3′ (R)</td></tr>
<tr><td colspan="2">EF-1α</td><td>5′-CCT TCA AAT ATG CCT GGG T-3′ (F)
5′-CAG CAC AGT CAG CTT GAG AG-3′ (R)</td></tr>
<tr><td colspan="2">GAPDH</td><td>5′-ACC TAC GCT CAG ATC AAA GAA G-3′ (F)
5′-CAT ACC AGG AGA TGA GCT TCA C-3′ (R)</td></tr>
<tr><td colspan="4">qPCR protocol</td></tr>
<tr><td colspan="2">Complete reaction conditions</td><td colspan="2">SYBR Green Master Mix (Roche)</td></tr>
<tr><td colspan="2">Reaction volume and amount of cDNA/DNA</td><td colspan="2">Reaction volume: 10 µl
Amount of cDNA: 1 µl</td></tr>
<tr><td colspan="2">Primer</td><td colspan="2">Primer: 0.5 mM of forward and reverse primer</td></tr>
<tr><td colspan="2">Polymerase, Mg^{2+}, dNTP, buffer</td><td colspan="2">Included in the SYBR Green Master Mix (Roche)</td></tr>
<tr><td colspan="2">Complete thermocycling parameters</td><td colspan="2">Universal cycling conditions:
10 min at 95 °C
40 cycles: 15 s at 95 °C and 60 s at 60 °C</td></tr>
</table>

Manufacturer of qPCR instrument	LightCycler® 96（Roche）
**qPCR validation**	
Specificity (gel, sequence, melt or digest)	Samples with a melt temperature (T_m) deviating from the product specific T_m were excluded
For SYBR Green I, Cq of the NTC	NTC of qPCR reaction gave no amplification
PCR efficiency	0.85-1.15
R^2 of calibration curve	≥ 99%
**Data analysis**	
qPCR analysis program (source, version)	The Rotor-Gene 5 software (Corbett Research, Rotor-Gene, Australia)
Method of Cq determination	LightCycler96_1.1.0（Roche）
Results for NTCs	NTC of qPCR reaction gave no amplification
Justification of number and choice of reference genes	geNorm and NormFinder analyses
Description of normalization method	$2^{-\Delta\Delta Cq}$ (27)
Statistical methods for results significance	ANOVA, Bonferroni test
